# The Role of miR-4256/HOXC8 Signaling Axis in the Gastric Cancer Progression: Evidence From lncRNA-miRNA-mRNA Network Analysis

**DOI:** 10.3389/fonc.2021.793678

**Published:** 2022-01-17

**Authors:** Haijuan Gu, Yuejiao Zhong, Jibin Liu, Qian Shen, Rong Wei, Haixia Zhu, Xunlei Zhang, Xianxian Xia, Min Yao, Meixin Ni

**Affiliations:** ^1^ Department of Pharmacy, Tumor Hospital Affiliated to Nantong University, Nantong, China; ^2^ Department of Medical Oncology, The Affiliated Cancer Hospital of Nanjing Medical University & Jiangsu Cancer Hospital & Jiangsu Institute of Cancer Research, Nanjing, China; ^3^ Institute of Oncology, Tumor Hospital Affiliated to Nantong University, Nantong, China; ^4^ Department of Oncology, Tumor Hospital Affiliated to Nantong University, Nantong, China; ^5^ Clinical Laboratory, Tumor Hospital Affiliated to Nantong University, Nantong, China

**Keywords:** gastric cancer, network, overall survival, miR-4256, HOXC8, proliferation, migration

## Abstract

Gastric cancer is a deadly human malignancy and the molecular mechanisms underlying gastric cancer pathophysiology are very complicated. Thus, further investigations are warranted to decipher the underlying molecular mechanisms. With the development of high-throughput screening and bioinformatics, gene expression profiles with large scale have been performed in gastric cancer. In the present study, we mined The Cancer Genome Atlas (TCGA) database and analyzed the gene expression profiles between gastric cancer tissues and normal gastric tissues. A series of differentially expressed lncRNAs, miRNAs and mRNAs between gastric cancer tissues and normal gastric tissues were identified. Based on the differentially expressed genes, we constructed miRNA-mRNA network, lncRNA-mRNA network and transcriptional factors-mRNA-miRNA-lncRNA network. Furthermore, the Kaplan survival analysis showed that high expression levels of EVX1, GBX2, GCM1, HOXC8, HOXC9, HOXC10, HOXC11, HOXC12 and HOXC13 were all significantly correlated with shorter overall survival of the patients with gastric cancer. On the other hand, low expression level of HOXA13 was associated with shorter overall survival of patients with gastric cancer. Among these hub genes, we performed the *in vitro* functional studies of HOXC8 in the gastric cancer cells. Knockdown of HOXC8 and overexpression of miR-4256 both significantly repressed the gastric cancer cell proliferation and migration, and miR-4256 repressed the expression of HOXC8 *via* targeting its 3’ untranslated region in gastric cancer cells. Collectively, our results revealed that a complex interaction networks of differentially expressed genes in gastric cancer, and further functional studies indicated that miR-4256/HOXC8 may be an important axis in regulating gastric cancer progression.

## Introduction

Gastric cancer is one of the deadly human malignancies, has become a health burden in the world, and is specifically prevalent in China (1–3). In the early stages of gastric cancer, the symptomatology of this disease is non-specific and rare. Surgical resection and/or chemotherapy are the main strategies for the treatment of gastric cancer, however, the 5-year overall survival of patients with gastric cancer is less than 20% and the prognosis among these patients remains poor (4–6). Therefore, further exploring the underlying molecular mechanisms of gastric cancer progression will be beneficial for effective management for patients with gastric cancer.

The non-coding RNAs including long non-coding RNAs (lncRNAs) and microRNAs (miRNAs) have no capacity for coding proteins, and have been a hot research topic in recent years (7–9). LncRNAs are a type of non-coding RNA with more than 200 nucleotides in length, and miRNAs belong to a class of small non-coding RNAs with ~21 nucleotides in length (10). Studies have proved that lncRNAs and miRNAs can play a crucial role in the majority of key cellular processes involved in the maintenance of cellular homeostasis by regulating various molecular mechanisms (11). Various studies have demonstrated that both lncRNAs and miRNAs involve in the pathophysiology of gastric cancer. Xu et al., revealed a positive feedback loop of lncRNA-plasmacytoma variant translocation 1 and forkhead box protein M1 that could promote gastric cancer growth and invasion (12). In the development and treatment of many human diseases, the regulatory roles between lncRNAs and miRNAs are important (13). Tian et al., showed that SP1-activated gastric cancer metastasis-associated lncRNA functions as a competing endogenous RNA to promote tumor metastasis by sponging miR-124 and miR-34a in gastric cancer (14). Zhang et al., showed that lncRNA small nucleolar RNA host gene 8 promoted proliferation and invasion of gastric cancer cells by targeting the miR-491/platelet-derived growth factor receptor alpha axis (15). Luo et al., s showed that lncRNA LINC00483 promotes gastric cancer development through regulating mitogen-activated protein kinase 1 expression by sponging miR-490-3p (16). Studies also demonstrated that transcriptional factors (TFs) are key modulators involved in regulating transcription and post-transcriptional regulation of genes through binding to particular DNA sequences, and involves in various biological processes (17, 18). Therefore, more investigations are warranted to interpret the interrelationships of lncRNAs, miRNAs, mRNAs, and TFs in gastric cancer.

With the advancement in high-throughput screening and bioinformatics, more and more large-scale genes (lncRNAs, miRNAs and mRNAs) have been identified by using gene expression profiles analysis. In this study, we explored the mRNA, lncRNA and miRNA gene expression profile from The Cancer Genome Atlas (TCGA) database by using gastric cancer samples and normal gastric samples. Based on the differentially expressed miRNAs, lncRNAs and mRNAs, we performed the functional enrichment analysis and constructed miRNA-mRNA network, lncRNA-mRNA network and TFs-mRNA-miRNA-lncRNA network, respectively. Moreover, the prognostic potential of the hub genes in the study was evaluated using Kaplan-Meier survival analysis of gastric cancer patients. Furthermore, we also performed *in vitro* functional studies to determine the potential role of the miR-4256/HOXC8 axis in modulating the progression of gastric cancer. The current research may provide novel insights into exploring potential therapeutic targets for gastric cancer.

## Materials and Methods

### Data Acquisition and Analysis of Differentially Expressed Genes

The present study acquired the data from The Cancer Genome Atlas (TCGA) (https://cancergenome.nih.gov/) including RNA‐Seq data and clinical characteristics of the cancer patients. In the study, we included RNA-Seq data containing 376 gastric cancer tissues and 32 para-cancerous tissues. To compare the normal group with the gastric cancer group, the “edgeR” package in R software was used to detect the differentially expressed RNA (including mRNA, lncRNA, and miRNA) by using the cut-off parameters of |log2foldChange(FC)| > 1 and the false discovery rate (FDR) P‐value < 0.05.

### Functional Enrichment Analysis

Gene ontology (GO) enrichment analyses in terms of biological process (BP), molecular function (MF), and cellular component (CC) for differentially expressed genes was performed by using the BiNGO tool in Cytoscape (19).

Gene Set Enrichment Analysis was performed based on mRNA-seq data using the GSEA software version 3.02. The mRNA-seq data were pre-ranked to form a gene list according to their expression levels between two developmental stages. Following this, certain gene sets from MSigDB were mapped to the pre-ranked gene-list to calculate the enrichment score, in which 1000 permutations were used to calculate significance, and a certain gene set with FDR < 0.01 was considered significant.

### Survival Analysis

The association between hub genes and the overall survival of patients with gastric cancer was analyzed using the Kaplan-Meier Survival analysis. We collected the clinical characteristics of patients with gastric cancer from the TCGA database, the association between expression levels of different hub genes and the overall survival was performed using the “survival” package in R, and P < 0.05 was considered statistically significant.

### Construction of miRNA-mRNA Network, lncRNA-mRNA Network and TFs-mRNA-miRNA-lncRNA Network

Based on differentially expressed lncRNAs, mRNAs, and miRNAs. The lncRNA‐miRNA interaction relationship was obtained from the miRcode database (http://www.mircode.org/). The miRNA‐mRNA interactions were obtained from miRDB (http://www.mirdb.org/). The mRNA-mRNA and TFs-mRNA interactions were obtained from StringDB. The lncRNA‐mRNA co-expression relationship was obtained by the WGCNA algorithm. The co-expression correlation coefficient ≥ 0.7 is considered a significant correlation. MiRNA-mRNA network, lncRNA-mRNA network and TFs-mRNA-miRNA-lncRNA network were visualized by using the Cytoscape software. For the constructed network, nodes and edges were used to represent large biological data in an intuitive way, in which each node represents a biological molecule, the edges stand for the interactions between nodes, and the node degrees indicate the number of edges linked to a given node were calculated to exploit the hub nodes that possess essential biological functions.

### Cell Culture

Normal gastric cell lines (GES-1) and the gastric cancer cell lines (BGC-823 and AGS) were obtained from the ATCC (Manassas, USA), and cultured in DMEM medium (Gibco, Grand Island, USA) containing 10% fetal bovine serum (FBS, Gibco), 100 μg/ml streptomycin and 100 U/ml penicillin at 37°C in a 5% CO2 incubator.

### SiRNAs, miRNAs and Transfections

SiRNAs targeting HOXC8 and the control siRNA were synthesized by Genepharma (Shanghai, China). Mimics of miR-4256 and the negative control (NC) miRNA (mimics NC) were synthesized by RiboBio (Guangzhou, China). SiRNAs, miRNA mimics were transfected into cells using Lipofectamine 2000 (Invitrogen, USA). The experiment was performed according to the manufacturer’s instructions.

### Quantitative Real-Time PCR

Total RNA from cells was extracted using TRIzol reagent (Invitrogen), and total RNA was reversely transcribed into complementary DNA (cDNA) by using the PrimeScript RT Reagent Kit (Takara). qPCR was performed using SYBR Premix Ex Taq™ (TakaRa) on an ABI7900 real-time PCR detection system (Applied Biosystems, Foster City, USA) following the manufacturer’s instructions. GAPDH and U6 were respectively used as an internal control for HOXC8 and miR-4256 expression. The relative levels of genes were determined using the 2^−ΔΔCt^ method.

### Cell Counting Kit-8 (CCK-8) Assay

Cell viability was detected by using the CCK-8 assay (Beyotime, Shanghai, China). Cells with respective treatments were seeded into 96-well plates at 2 × 10^3^ cells/well cell concentration. After cell culture for 0 h, 24 h, 48 h, 72 h respectively, 10 μL CCK-8 solution was added to each well and incubated at 37°C for 2 h. The absorbance was measured at a wavelength of 450 nm for each well by using a microplate reader (BioTek Instruments, Winooski, USA).

### EdU Incorporation Assay

For EdU incorporation assays, cells with respective treatments were incubated with 20 μM EdU (RiboBio, Guangzhou, China) according to the manufacturer’s instructions. After that, cells were fixed with 4% paraformaldehyde (Sigma-Aldrich, St. Louis, USA) for 10 min and permeabilized with 0.2% Triton-100 for 15 min at room temperature. Subsequently, the cells were stained with Apollo solution and Hoechst 33342. Finally, random fields were selected, and the numbers of proliferative cells were evaluated by fluorescence microscopy.

### Wound Healing Assay

Gastric cancer cells with respective treatments were cultured in six-well plates at 3°C. Scratch wounds were created by using the fine end of 200 μL pipette tips. Images of migrated cells were captured under microscopy at indicated time points (0 h after wound scratching and 24 h after wound scratching). The % of wound healing was calculated using the formula below: wound healing (%) = (wound width at 0 h – wound width at 24 h)/wound width at 0 h X 100%.

### Luciferase Reporter Assay

The fragments of wild type 3’UTR of HOXC8 that were targeted by miR-4256 were amplified from the genome and were subcloned into the pGL3 vector (Promega, Madison, USA). The corresponding mutation of the reporter vector containing 3’UTR of HOXC8 was generated by the site-directed mutagenesis. BGC-823 and AGS cells were seeded in 24-well plates (3 × 10^4^ cells/well). Mimics NC or miR-4256 mimics were co-transfected with the reporter plasmid into gastric cancer cells using Lipofectamine 2000. After 48 h, the cells were collected and lysed with passive lysis buffer. The luciferase activities were assessed using the Dual-Luciferase Assay Kit (Promega) according to the manufacturer’s protocol. Firefly luciferase activity was normalized to Renilla luciferase activity.

### Statistical Analysis

All the data were presented as mean ± standard deviation. Comparisons between different groups were analyzed with GraphPad Prism v7.0 software (GraphPad Software, La Jolla, USA) using an unpaired Student’s t-test or one-way ANOVA followed by Bonferroni’s multiple comparison tests. P < 0.05 was considered statistically significant.

## Results

### Differentially Expressed Genes in Gastric Cancer Tissues and Normal Gastric Tissues From the TGCA Database

**Figure 1:**
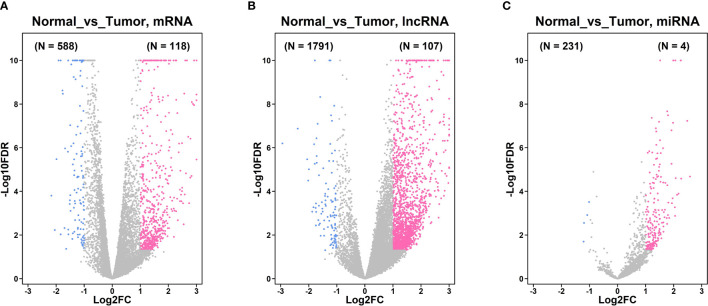
Differentially expressed genes in gastric cancer tissues and normal gastric tissues from TGCA database. **(A)** Volcano plot of the differentially expressed mRNA between gastric cancer tissues and normal gastric tissues. **(B)** Volcano plot of the differentially expressed lncRNA between gastric cancer tissues and normal gastric tissues. **(C)** Volcano plot of the differentially expressed miRNA between gastric cancer tissues and normal gastric tissues. Pink dots represent up-regulated mRNA or lncRNAs; blue dots represent down-regulated mRNA or lncRNAs; grey dots represent non-significantly expressed mRNA or lncRNAs.

Based on the analysis of the TGCA database, a total of 376 gastric cancer tissues and 32 adjacent normal gastric tissues were included in the analysis. As shown in **Figure 1A**, a total of 118 up-regulated mRNAs and 588 down-regulated mRNAs were detected between gastric cancer tissues and normal gastric tissues. A total of 107 up-regulated lncRNAs and 1791 lncRNAs were identified between gastric cancer tissues and normal gastric tissues (**Figure 1B**). Furthermore, a total of 231 up-regulated and 4 down-regulated miRNAs were detected between gastric cancer tissues and normal gastric tissues (**Figure 1C**).

### GO Enrichment and GSEA Analysis of Differentially Expressed mRNAs

**Figure 2:**
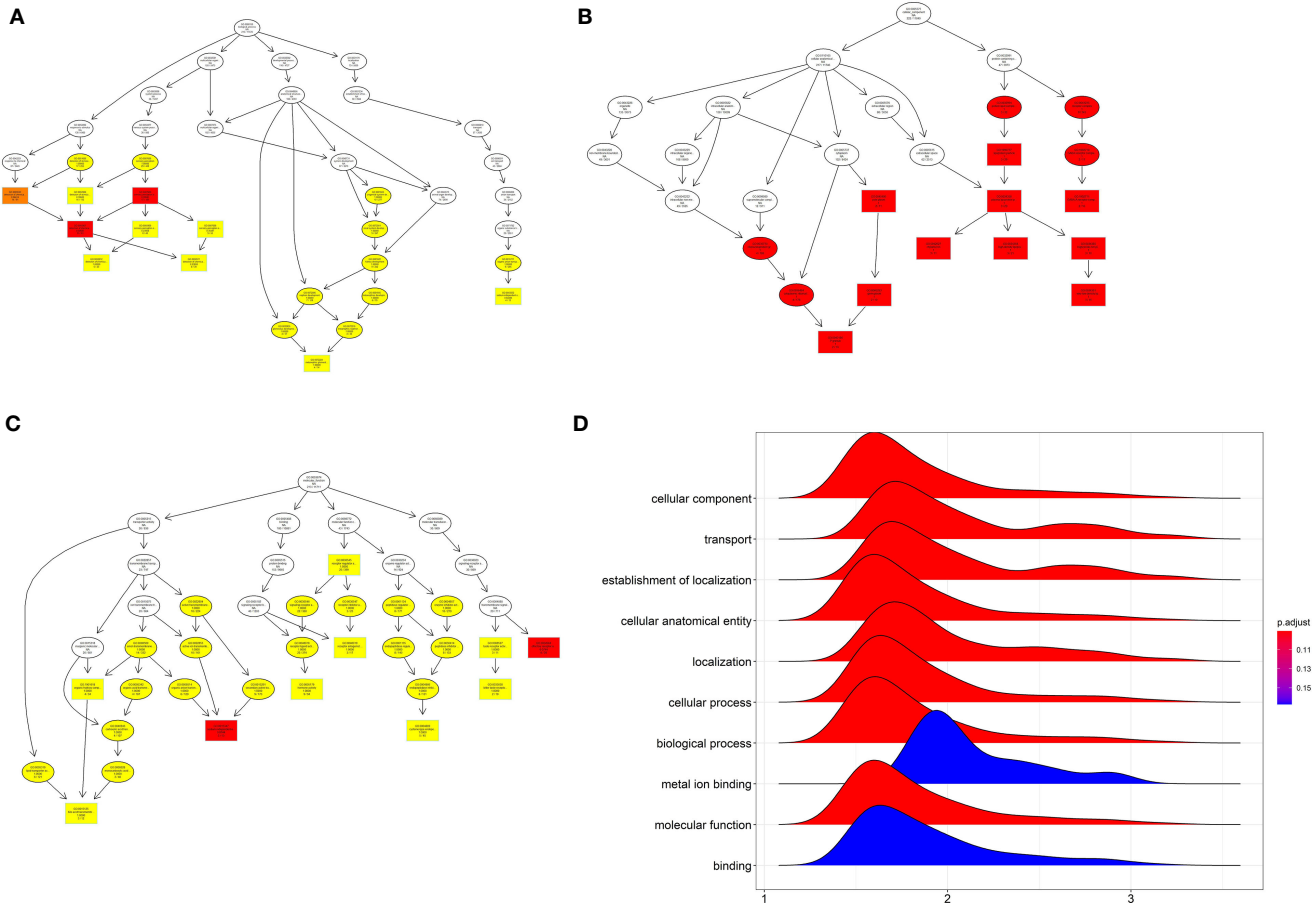
GO enrichment analysis and GSEA of differentially expressed mRNA. **(A)** The GO_BP enrichment analysis of differentially expressed mRNA. **(B)** The GO_MF enrichment analysis of differentially expressed mRNA. **(C)** The GO-CC enrichment analysis of differentially expressed mRNA. **(D)** GSEA analysis of differentially expressed mRNA.

For the GO_biological process enrichment analysis, the mRNAs were mainly enriched in “sensory perception”, “system development” and so on (**Figure 2A**). For the GO_cellular component analysis, the mRNAs were mainly enriched in “pole plasm”, “germ plasm”, “high density lipoprotein” and so on (**Figure 2B**). For the GO_molecular function analysis, the mRNAs were mainly enriched in “hormone activity”, “transmembrane transport” and so on (**Figure 2C**). The GSEA analysis further revealed that the mRNAs were mainly enriched in “cellular component”, “transport”, “establishment of localization”, “cellular anatomical entity”, “localization”, “cellular process” and etc (**Figure 2D**).

**Figure 3:**
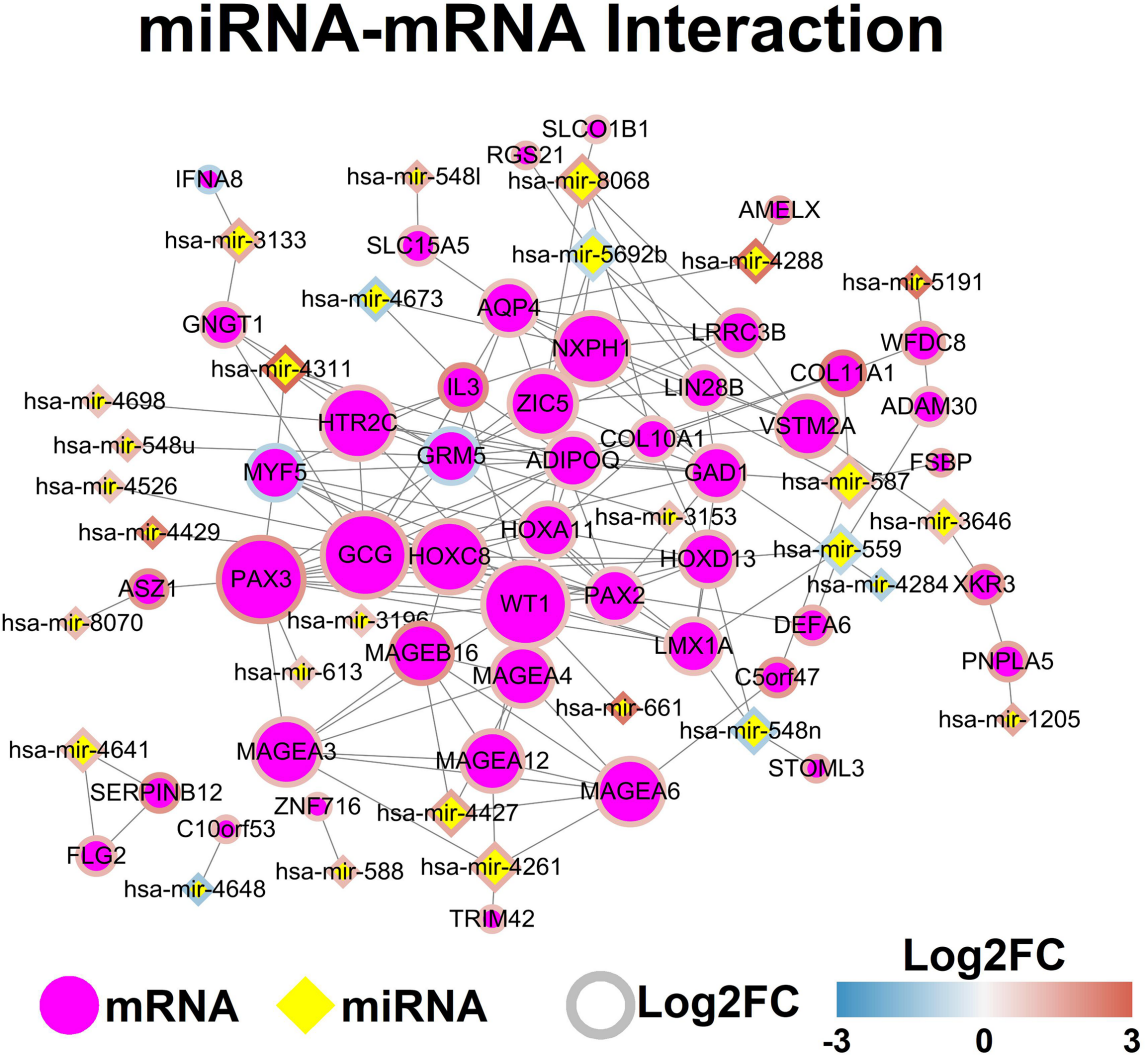
MiRNA-mRNA network. Each node represents one genes, and ach edge represents the interaction between genes. MiRNAs and mRNAs are indicated using diamond shapes and circle shapes respectively.

The potential miRNA and mRNA interaction was predicted by using the miRDB database, and the mRNA interaction network was constructed by using StringDB database. The miRNA-mRNA network was shown in **Figure 3**.

**Figure 4:**
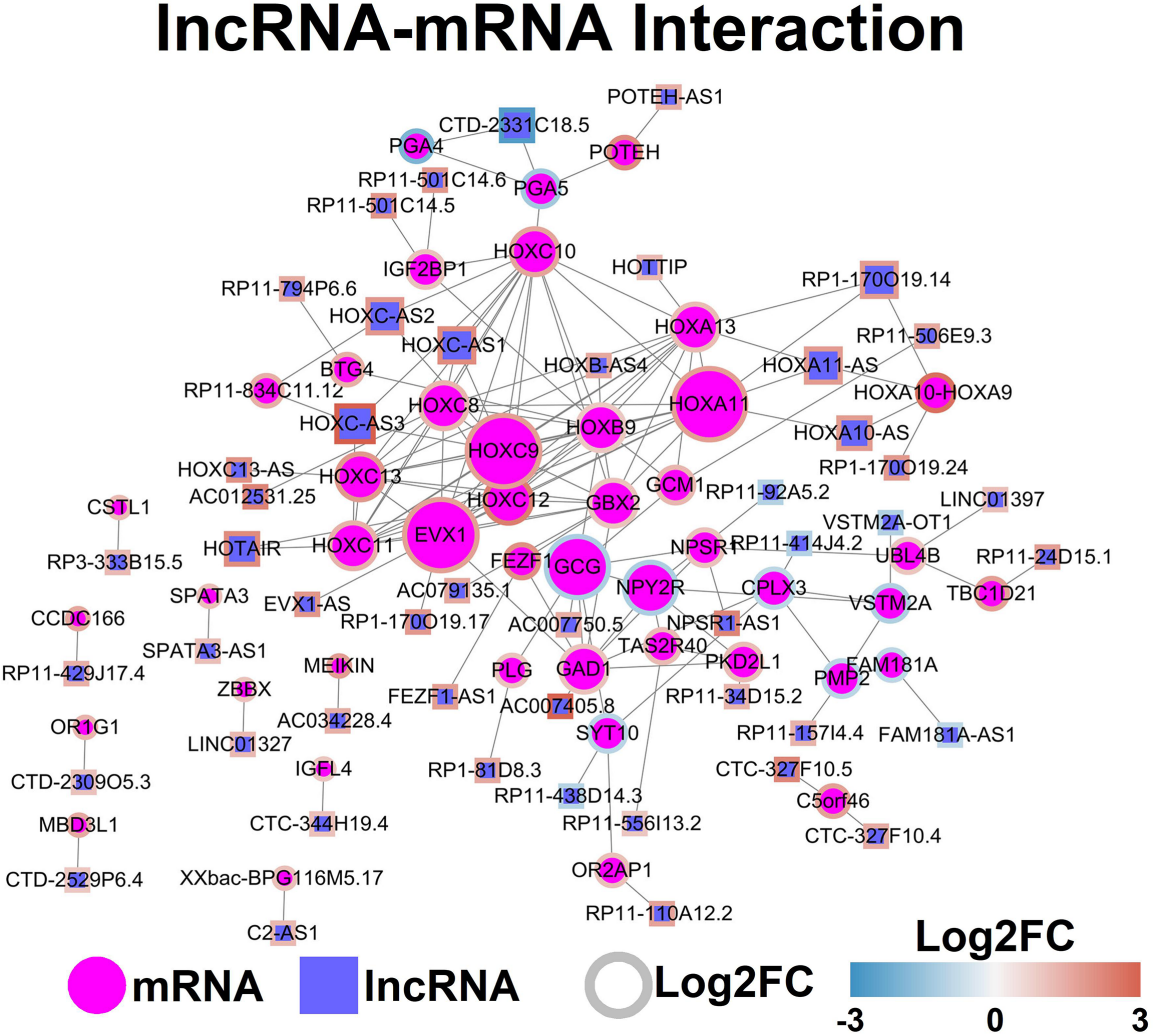
LncRNA-mRNA network. Each node represents one gene, and each edge represents the interaction between genes. LncRNAs and mRNAs are indicated using circular shapes and circle shapes respectively.

The potential interaction between lncRNAs and mRNAs was predicted by analyzing the mRNA in the lncRNA-located chromosome (+/- kb), and the mRNA interaction network was constructed by using StringDB database. The lncRNA-mRNA network was demonstrated in **Figure 4**.

**Figure 5:**
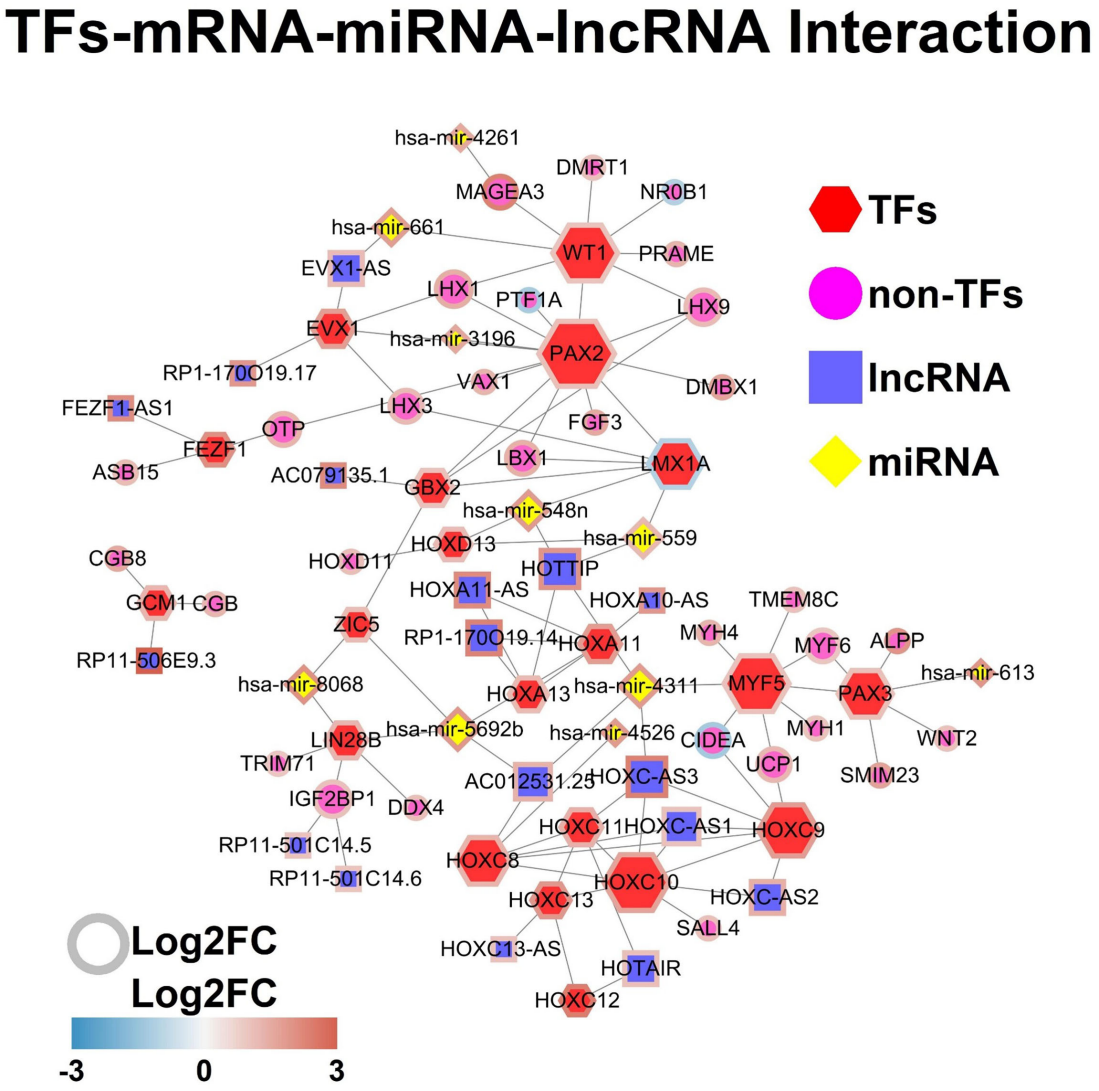
TFs-mRNA-miRNA-lncRNA network. Each node represents one gene, and each edge represents the interaction between genes. TFs, non-TFs, lncRNAs and miRNAs are indicated using hexagon, circular, square and diamond shapes, respectively.

The TFs-mRNA-miRNA-lncRNA network was constructed by using the StringDB database, and the TFs-mRNA-miRNA-lncRNA network was shown in **Figure 5**.

### GO and GSEA Analysis of All Differentially Expressed Genes

**Figure 6:**
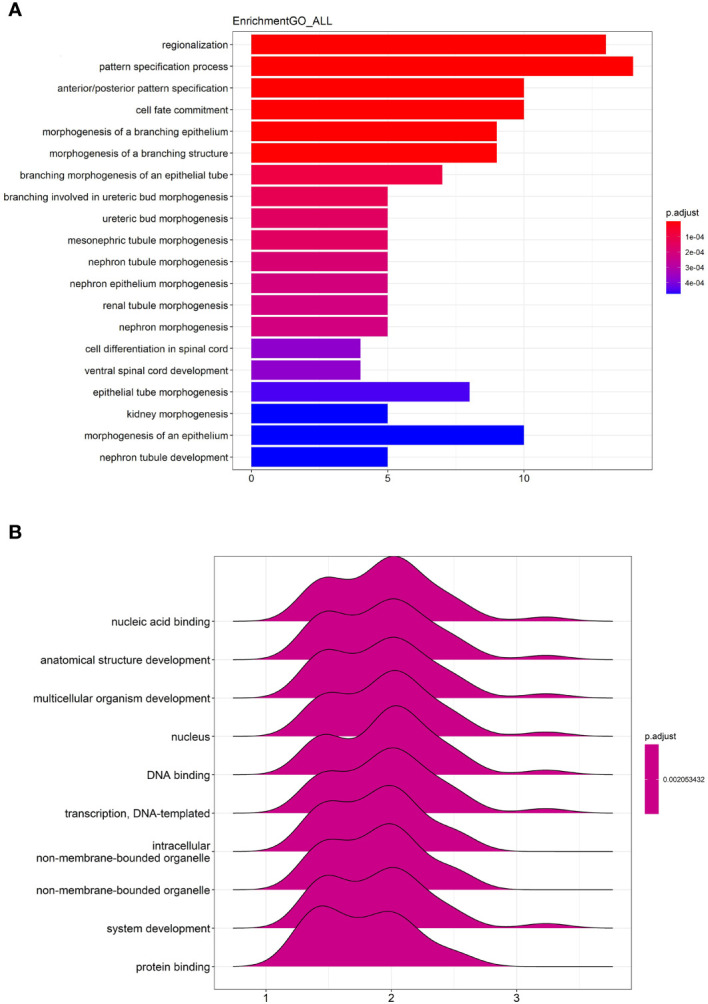
GO enrichment and GSEA analysis of all differentially expressed genes. **(A)** The top 20 most significant changes in the GO enrichment analysis. **(B)** The top 10 most significant changes in the GSEA enrichment analysis.

The GO enrichment analysis of all differentially expressed genes showed that the genes were significantly enriched in “regionalization”, “pattern specification process”, “anterior/posterior pattern specification”, “cell fate commitment”, “morphogenesis of a branching epithelium” and etc (**Figure 6A**).

The GSEA analysis of all differentially expressed genes were shown in **Figure 6B**. The genes were mainly enriched in “nucleic acid binding”, “anatomical structure development”, “multicellular organism development”, “nucleus”, “DNA binding”, “transcription, DNA-templated”, “intracellular non-membrane-bounded organelle”, “non-membrane-bounded organelle”, “system development” and “protein binding”.

### Kaplan Survival Analysis for the Association of Gene Expression Levels and the Overall Survival of Patients With Gastric Cancer

**Figure 7:**
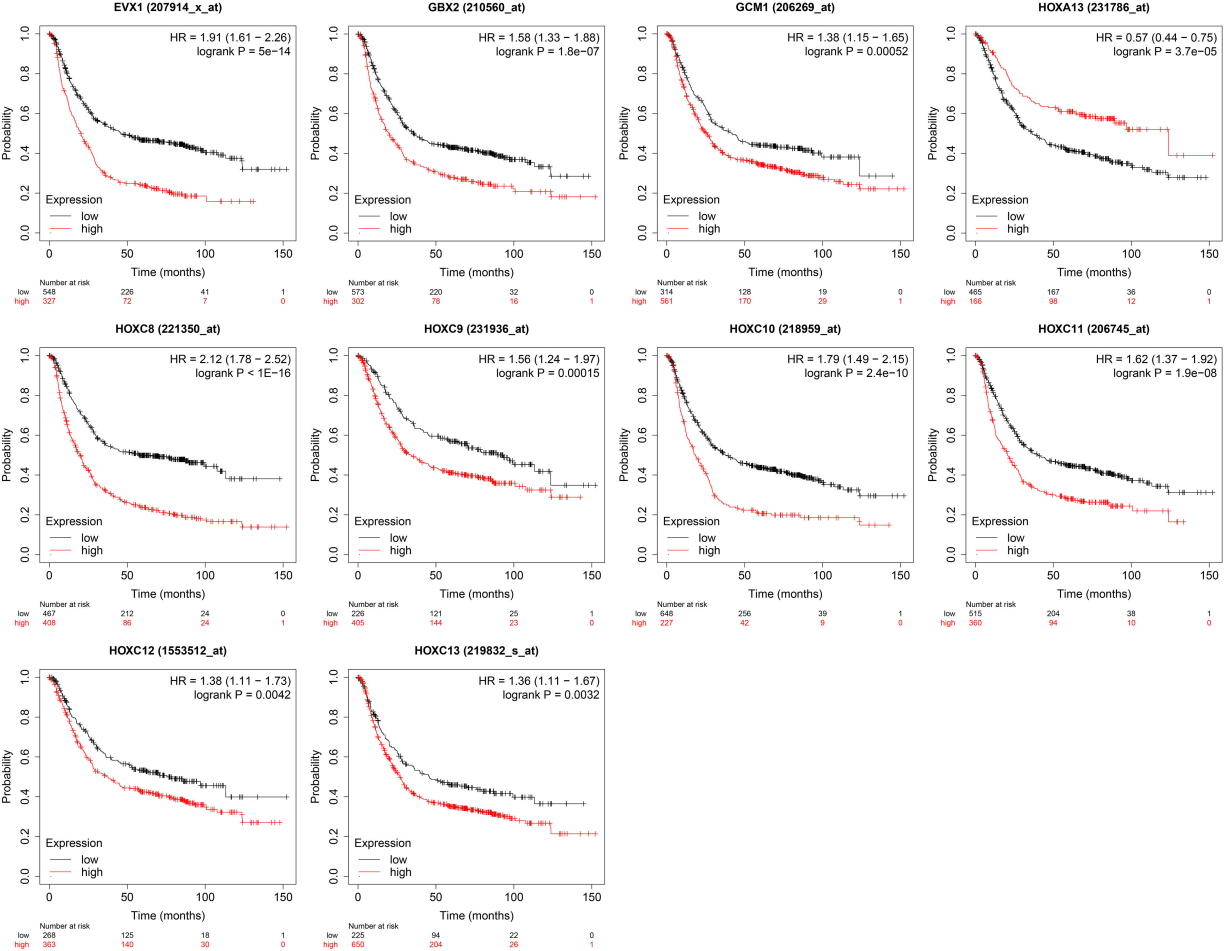
Kaplan survival analysis for the association of gene expression levels and the overall survival of patients with gastric cancer. The genes including EXV1, GBX2, GCM1, HOXA13, HOXC8, HOXC9, HOXC10, HOXC11, HOXC12 and HOXC13 were chosen for the survival analysis.

**Figure 8:**
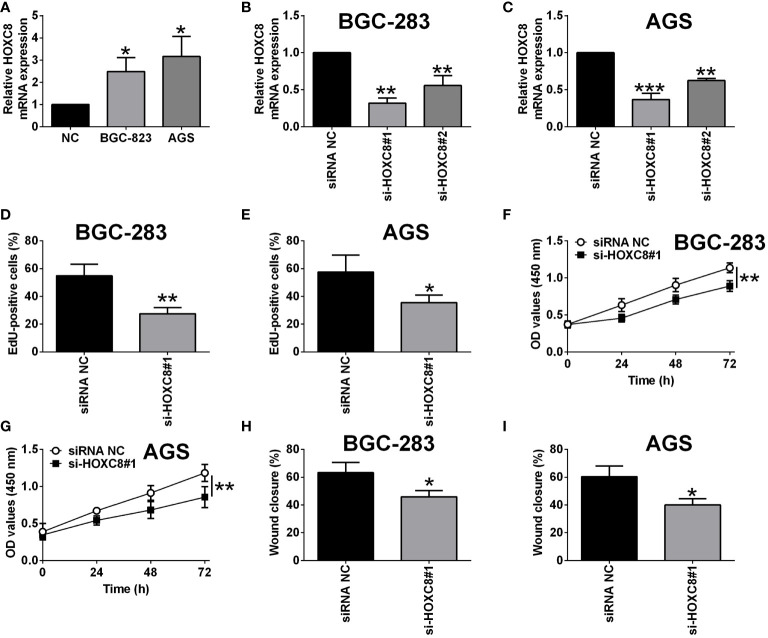
Knockdown of HOXC8 suppressed gastric cancer cell proliferation and migration. **(A)** HOXC8 mRNA expression level in GES-1, BGC-823 and AGS cells was determined by qRT-PCR. **(B, C)** BGC-823 cells and AGS were respectively transfected with HOXC8 siRNAs or the corresponding negative controls, and at 24 h after transfection, the relative mRNA expression of HOXC8 was determined by qRT-PCR. **(D-I)** BGC-823 cells and AGS were respectively transfected with HOXC8 siRNAs or the corresponding negative controls, at 24 h after transfection, the cell proliferation of BGC-823 **(D)** and AGS **(E)** was determined by EdU incorporation assay; the cell viability of BGC-823 **(F)** and AGS **(G)** was determined by CCK-8 assay; the cell migration of BGC-823 and AGS; the cell migration of BGC-823 **(H)** and AGS **(I)** was determined by wound healing assay. N = 3. *P < 0.05, **P < 0.01 and ***P < 0.001.

The association between the gene expression levels and overall survival of the patients with gastric cancer was further analyzed using the TGCA database. As shown in **Figure 8**, high expression levels of EVX1, GBX2, GCM1, HOXC8, HOXC9, HOXC10, HOXC11, HOXC12 and HOXC13 were all significantly correlated with shorter overall survival of the patients with gastric cancer. On the other hand, a low expression level of HOXA13 was associated with shorter overall survival of patients with gastric cancer (**Figure 7**).

### Knockdown of HOXC8 Suppressed Gastric Cancer Cell Proliferation and Migration

Based on the literature research, several members of the HOXC family have been well studied for their role in gastric cancer (20, 21), however, HOXC8 has not been fully elucidated for its role in regulating gastric cancer progression. Thus, we firstly examined if HOXC8 was dysregulated in the gastric cancer cells. As shown in **Figure 8A**, the mRNA expression of HOXC8 was up-regulated in the gastric cancer cells when compared to GES-1 cells. To determine the effects of HOXC8 on gastric cancer cell proliferation and migration, we performed the loss-of-function assay. The knockdown of HOXC8 was carried out in both BGC-823 and AGS cells by transfecting these cells with HOXC8 siRNAs. HOXC8 siRNAs transfection significantly down-regulated the mRNA expression of HOXC8 in both BGC-823 and AGS cells when compared to siRNA control transfection (**Figures 8B, C**). The EdU incorporation assay was used to determine gastric cancer cell proliferation, and HOXC8 knockdown reduced the number of EdU-positive BGC-823 and AGS cells when compared to the control groups (**Figures 8D, E**). The CCK-8 assay results showed that the cell viability of BGC-823 and AGS cells was significantly attenuated by HOXC8 silence (**Figures 8F, G**).

### Overexpression of miR-4256 Down-Regulated HOXC8 Expression and Suppressed Gastric Cancer Cell Proliferation and Migration

**Figure 9:**
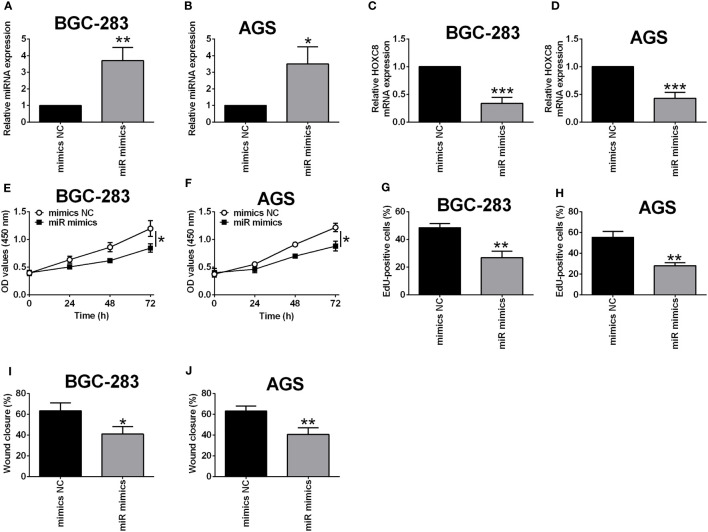
Overexpression of miR-4256 down-regulated HOXC8 expression and suppressed gastric cancer cell proliferation and migration. **(A-J)** BGC-823 cells and AGS were respectively transfected with HOXC8 siRNAs or the corresponding negative controls, and at 24 h after transfection, the relative expression of miR-4256 in BGC-823 **(A)** and AGS **(B)** cells was determined by qRT-PCR; the relative mRNA expression of HOXC8 in BGC-823 **(C)** and AGS cells **(D)** was determined by qRT-PCR; the cell proliferation of BGC-823 **(E)** and AGS **(F)** was determined by EdU incorporation assay; the cell viability of BGC-823 **(G)** and AGS **(H)** was determined by CCK-8 assay; the cell migration of BGC-823 and AGS; the cell migration of BGC-823 **(I)** and AGS **(J)** was determined by wound healing assay. N = 3. *P < 0.05, **P < 0.01 and ***P < 0.001.

**Figure 10:**
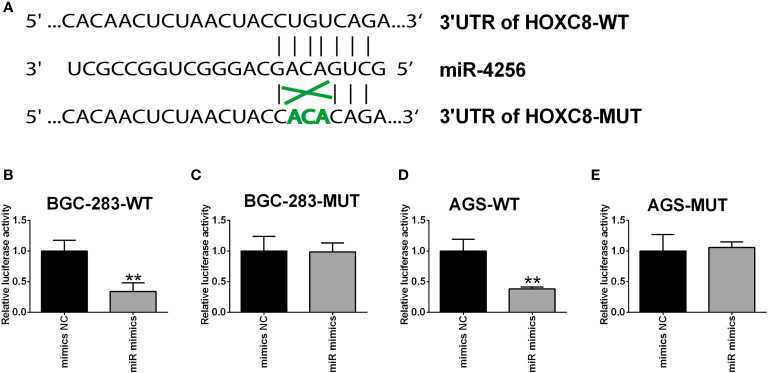
HOXC8 was a direct target of miR-4256. **(A)** The predicted binding sites between miR-4256 and HOXC8 3’UTR. **(B, C)** The relative luciferase activity in BGC-283 cells co-transfected with miR-4256 mimics (or mimics NC) and wide type **(B)** or mutant **(C)** 3’UTR of HOXC8. **(D, E)** The relative luciferase activity in AGS cells co-transfected with miR-4256 mimics (or mimics NC) and wide type **(D)** or mutant **(E)** 3’UTR of HOXC8. **P<0.01.

Based on the network analysis, miR-4256 showed potential interaction with HOXC8. Furthermore, we examined if miR-4256 could affect the expression of HOXC8 in BGC-823 and AGS cells. As shown in **Figures 10A, B**, miR-4256 mimics transfection significantly up-regulated miR-4256 expression in both BGC-283 and AGS cells when compared to mimics NC transfection (**Figures 9A, B**). The qRT-PCR assay further confirmed that miR-4256 overexpression repressed the mRNA expression level of HOXC8 in both BGC-823 and AGS cells (**Figures 9C, D**). The CCK-8 assay showed that the cell viability of BGC-823 and AGS cells was significantly attenuated by miR-4256 overexpression (**Figures 9E, F**). Consistently, miR-4256 overexpression inhibited the cell proliferation of BGC-823 and AGS cells (**Figures 9G, H**). Moreover, the wound healing assay revealed that miR-4256 mimics transfection enhanced the wound healing of gastric cancer cells when compared to mimics NC transfection (**Figures 9I, J**).

### HOXC8 Was a Direct Target of miR-4256

To further clarify the interaction between miR-4256 and HOXC8, the predicted binding sites between miR-4256 and HOXC8 3’UTR were predicted by the TargetScan online tool (**Figure 10A**). The luciferase reporter assay showed that miR-4256 overexpression repressed the luciferase activity of the reporter vector harboring the wild type 3’UTR of HOXC8 (**Figure 10B**), but not the mutant 3’UTR of HOXC8 (**Figure 10C**) in BGC-823 cells. Consistently, miR-4256 overexpression repressed the luciferase activity of the reporter vector harboring the wild type 3’UTR of HOXC8 (**Figure 10D**), but not the mutant 3’UTR of HOXC8 (**Figure 10E**) in AGS cells.

## Discussion

Gastric cancer is a deadly human malignancy and the molecular mechanisms underlying gastric cancer pathophysiology are very complicated (22). Thus, further investigations are warranted to decipher the underlying molecular mechanisms. With the development of high-throughput screening and bioinformatics, gene expression profiles with a large scale have been performed in gastric cancer. In the present study, we harnessed the TCGA database and analyzed the gene expression profiles between gastric cancer tissues and normal gastric tissues. A series of differentially expressed lncRNAs, miRNAs and mRNAs between gastric cancer tissues and normal gastric tissues were identified. Based on the differentially expressed genes, we constructed miRNA-mRNA network, lncRNA-mRNA network and TFs-mRNA-miRNA-lncRNA network. Furthermore, the Kaplan survival analysis showed that high expression levels of EVX1, GBX2, GCM1, HOXC8, HOXC9, HOXC10, HOXC11, HOXC12 and HOXC13 were all significantly correlated with shorter overall survival of the patients with gastric cancer. On the other hand, a low expression level of HOXA13 was associated with shorter overall survival of patients with gastric cancer. Among these hub genes, we performed the *in vitro* functional studies of HOXC8 in gastric cancer cells. Knockdown of HOXC8 and overexpression of miR-4256 both significantly repressed the gastric cancer cell proliferation and migration, and miR-4256 repressed the expression of HOXC8 *via* targeting its 3’UTR in gastric cancer cells. Collectively, our results revealed that complex interaction networks of differentially expressed genes in gastric cancer, and further functional studies indicated that miR-4256/HOXC8 may be an important axis in regulating gastric cancer progression.

The interaction networks of differentially expressed genes in gastric cancer have been revealed in various studies by using bioinformatics analysis. Zheng et al., performed the weighted correlation network analysis and constructed the tumor-specific mRNA-miRNA-lncRNA network in gastric cancer and developed a 12-gene signature from both prognosis-related and tumor-specific genes (23). Li et al., systematically analyzed the aberrantly expressed genes in human GC to construct a ceRNA network by using multiple bioinformatic tools and found that H19 promoted gastric carcinogenesis by sponging miR-miR-29a-3p (24). Zhao et al., performed a comprehensive analysis of survival-related lncRNAs, miRNAs and mRNAs and identified a competing endogenous RNA network in gastric cancer (25). In the present study, we analyzed the TCGA dataset and constructed miRNA-mRNA network, lncRNA-mRNA network and TFs-mRNA-miRNA-lncRNA network based on the differentially expressed genes. Based on the network construction, ten hub genes were found to be associated with the overall survival of patients with gastric cancer.

Among the hub genes, we further examined the *in vitro* functions of HOXC8 in the gastric cancer cells and found that HOXC8 was up-regulated in the gastric cancer cells, suggesting that HOXC8 may act as an oncogene in gastric cancer. The functional studies showed that knockdown of HOXC8 suppressed gastric cancer cell proliferation, viability and migration. In fact, the role of HOXC8 has been deciphered in other types of cancers. Liu et al., showed that HOXC8 promoted proliferation and migration *via* transcriptional up-regulation of TGFbeta1 in non-small cell lung cancer (26). Shah et al., demonstrated that HOXC8 regulated self-renewal, differentiation and transformation of breast cancer stem cells (27). Moreover, Gong et al., showed that HOXC8 could enhance MGP expression, which in turn promoted the proliferation, migration and epithelial-mesenchymal transition of triple-negative breast cancer (28). Li et al., showed that HOXC8-dependent cadherin 11 expression facilitated breast cancer cell migration through Trio and Rac (29). On the other hand, expression of HOXC8 is inversely correlated with the progression and metastasis of pancreatic ductal adenocarcinoma (30); HOXC8 inhibited androgen receptor signaling in human prostate cancer cells by inhibiting SRC-3 recruitment to direct androgen target genes (31). Consistently, these results implied that HOXC8 may act as an oncogene to promote the progression of gastric cancer cells.

Based on the network analysis, we found that miR-4256 was a potential interactor for HOXC8. By confirming the interaction using TargetScan tool, we found that miR-4256 could target the 3’UTR of HOXC8. In fact, HOXC8 could be targeted by several miRNAs in the cancer studies. Mueller et al., revealed that miR-196a regulated melanoma-associated genes by regulating HOXC8 expression (32). Li et al., found that ratio of miR-196s to HOXC8 messenger RNA correlated with breast cancer cell migration and metastasis (33). HOXC8 could be targeted by miR-196a-5p and promote the cell growth of osteosarcoma (34). Previous studies also demonstrated that miR-4256 was down-regulated in rhabdomyosarcoma and renal cell carcinoma (35, 36). Consistently, our results showed that miR-4256 could negatively regulate the expression of HOXC8 in gastric cancer cells, and suppressed the proliferation and migration of gastric cancer cells. Collectively, our results indicated that miR-4256 acts as a tumor suppressor in gastric cancer cells by repressing HOXC8. However, based on the online KM Plotter tool, we found that high expression of miR-4256 was correlated with better overall survival of patients with gastric cancer, but the results were not statistically significant (**Supplementary Figure S1**), and the prognostic role of miR-4256 in gastric cancer should be further explored by including more patients. Whether mir-4256 can be used as a potential target for the prevention and treatment of gastric cancer should be further explored.

In conclusion, we identified the complex interaction networks in gastric cancer by analyzing TGCA database. Furthermore, ten hub genes have been identified to be associated with the overall survival of patients with gastric cancer. The functional studies indicated that HOXC8 may exert oncogenic effects on gastric cancer. Additional functional assays indicated that a novel miR-4256/HOXC8 signaling axis may involve the progression of gastric cancer.

## Data Availability Statement

The original contributions presented in the study are included in the article/**Supplementary Material**. Further inquiries can be directed to the corresponding author.

## Author Contributions

HG, YZ and MN conceptualized and designed the study. HG, JL, QS, RW, HZ, and XZ contributed to data collection. XX and MY analyzed and interpreted data. MN drafted the manuscript. All authors approved the final version of the manuscript.

## Funding

This study was supported by Grants from the Nantong Science and Technology Project (JCZ20165), Funding for the Health Commission of Nantong City (MB2019020), Funding for Hospital Pharmacy of Jiangsu Pharmaceutical Association (A201929), and Scientific Research Project of Jiangsu Provincial Health Committee (Z2021056).

## Conflict of Interest

The authors declare that the research was conducted in the absence of any commercial or financial relationships that could be construed as a potential conflict of interest.

## Publisher’s Note

All claims expressed in this article are solely those of the authors and do not necessarily represent those of their affiliated organizations, or those of the publisher, the editors and the reviewers. Any product that may be evaluated in this article, or claim that may be made by its manufacturer, is not guaranteed or endorsed by the publisher.

## Supplementary Material

Supplementary Figure S1 The correlation between miR-4256 expression level and the overall survival of patients with gastric cancer. The analysis was performed by using the online tool (Kaplan-Meier Plotter; https://kmplot.com/analysis).
