# Supplementary figures and images for: The Role of miR-4256/HOXC8 Signaling Axis in the Gastric Cancer Progression: Evidence From lncRNA-miRNA-mRNA Network Analysis
Source: Front Oncol. 2022 Jan 17;11:793678. doi: 10.3389/fonc.2021.793678 (PMC8801578; doi:10.3389/fonc.2021.793678)

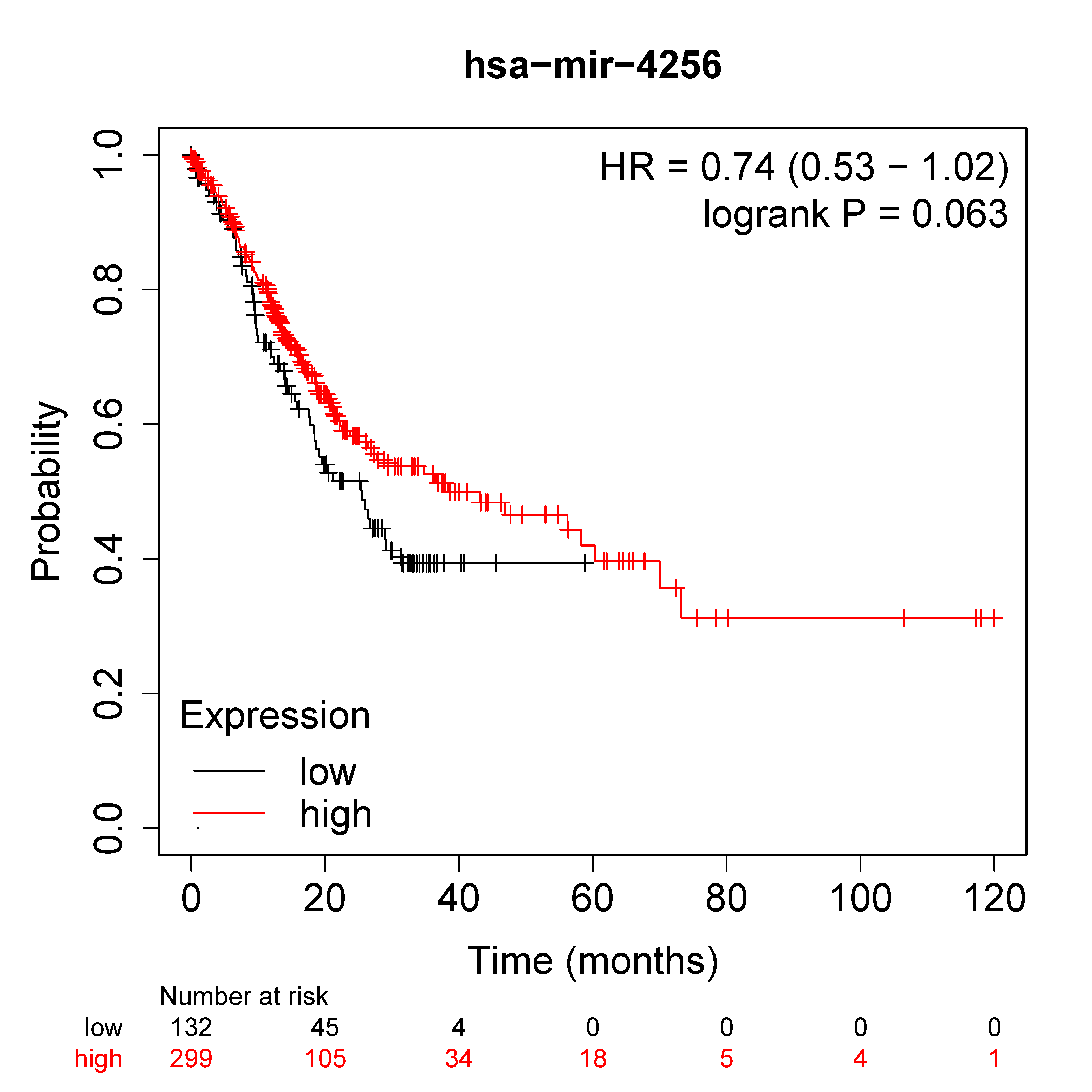

Supplement: Supplementary Figure S1 — The correlation between miR-4256 expression level and the overall survival of patients with gastric cancer. The analysis was performed by using the online tool (Kaplan-Meier Plotter; https://kmplot.com/analysis). [file Image_1.tiff]
